# CIRCE: a scalable Python package to predict cis-regulatory DNA interactions from single-cell chromatin accessibility data

**DOI:** 10.1093/bioinformatics/btag092

**Published:** 2026-02-24

**Authors:** Rémi Trimbour, Julio Saez-Rodriguez, Laura Cantini

**Affiliations:** Institut Pasteur, Université Paris Cité, CNRS UMR 3738, Machine Learning for Integrative Genomics Group, Paris F-75015, France; Heidelberg University, Faculty of Medicine, and Heidelberg University Hospital, Institute for Computational Biomedicine, Heidelberg, Germany; European Molecular Biology Laboratory, European Bioinformatics Institute (EMBL-EBI), Hinxton, Cambridgeshire, United Kingdom; Institut Pasteur, Université Paris Cité, CNRS UMR 3738, Machine Learning for Integrative Genomics Group, Paris F-75015, France

## Abstract

**Motivation:**

Chromatin 3D folding creates numerous DNA interactions, participating in gene expression regulation. Single-cell chromatin-accessibility assays now profile hundreds of thousands of cells, challenging existing methods for mapping cis-regulatory interactions.

**Results:**

We present CIRCE, a fast and scalable Python package to predict cis-regulatory DNA interactions from single-cell chromatin accessibility data. CIRCE re-implements the Cicero workflow to analyse single-cell atlases, cutting runtime and memory use by several orders of magnitude. We also provide new options to compute metacells, grouping similar cells to reduce data sparsity. We benchmarked CIRCE against Cicero on two datasets of different sizes and demonstrated the improvement from CIRCE’s metacells’ strategy with promoter capture Hi-C data. We also evaluated how DNA interaction predictions are impacted by different pre-processing. We observed a negative impact of Cicero’s count normalization, and the best performance was obtained with the single-cell count matrix directly. Finally, we demonstrated the scalability of CIRCE by processing a dataset of more than 700 000 cells and 1 million DNA regions in less than an hour. CIRCE should greatly facilitate the prediction of DNA region interactions for scverse and Python users, while providing new and up-to-date pre-processing insights.

**Availability and implementation:**

CIRCE is released as an open-source software under the AGPL-3.0 licence. The package source code is available on GitHub at https://github.com/cantinilab/CIRCE, and its documentation is accessible at https://circe.readthedocs.io. The code to reproduce the presented results is available as a Snakemake pipeline at https://github.com/cantinilab/circe_reproducibility.s.

## 1 Introduction

Cis-regulatory elements can regulate genes located hundreds of thousands of base pairs away through DNA folding, which brings distal regulatory elements into close spatial arrangement with the gene promoter region to facilitate interactions (Dekker *et al.* 2002, Lieberman-Aiden *et al.* 2009). The regulation of gene expression results from complex and dynamic interactions between all these regulatory regions.

Single-cell Assay for Transposase-Accessible Chromatin using sequencing (scATAC-seq) is a powerful technique for studying chromatin accessibility in individual cells. The chromatin accessibility profiles measured allow understanding the role of specific DNA elements, such as enhancers, to define cell state heterogeneity and regulate gene expression (Badia-i-Mompel *et al.* 2023).

While single-cell ATAC-seq provides insights into chromatin accessibility and potential interactions with transcription factors and the transcriptional machinery, identifying regulatory and interacting regions from chromatin accessibility remains challenging, as it does not inform about DNA conformation.

Several methods took interest in this challenge and proposed to infer gene–enhancer connections from single-cell ATAC-seq data alone (Pliner *et al.* 2018, Granja *et al.* 2021, Stuart *et al.* 2021, Seufert *et al.* 2024), single-cell multi-omics data (Zhang *et al.* 2022, Bravo González-Blas *et al.* 2023, Sakaue *et al.* 2024, Trimbour *et al.* 2024), or combining ATAC with other input data types, such as Hi-C data or DNA sequences (Fulco *et al.* 2019, Grover *et al.* 2024, Sheth *et al.* 2024). Among them, Cicero, an algorithm developed for analysing single-cell ATAC-seq data (Pliner *et al.* 2018), has been widely adopted to uncover cis-regulatory DNA interactions and gene regulatory mechanisms (Kamimoto *et al.* 2023, Trimbour *et al.* 2024). It aims to construct a global map of cis-regulatory interactions, considering the distance between regions and the technical effects in measurements.

Cicero was developed when single-cell datasets were relatively small and optimal preprocessing strategies had not been well established. Cicero shows limited resource usage performances when applied to very large high-resolution datasets produced nowadays (e.g. hundreds of thousands of cells) (Cusanovich *et al.* 2018, Yao *et al.* 2021, Zhang *et al.* 2021). Its single-cell preprocessing also relies by default on a clustering of cells from UMAP/t-SNE spaces, which do not accurately preserve distances between observations (Kobak and Linderman 2021, Chari and Pachter 2023). Furthermore, Cicero is only available as an R package, and integrating it in a Python workflow, in particular in the broadly used scverse ecosystem, requires non-standard hybrid environments and additional effort. For example, both gene regulatory network inference methods, HuMMuS (Trimbour *et al.* 2024) and CellOracle (Kamimoto *et al.* 2023), are currently based on Cicero outputs and require users to export them to follow the next Python workflow steps.

To overcome these limitations, we introduce CIRCE, a fast and scalable Python package to analyse single-cell ATAC datasets and atlases. We update the metacells computation strategy and preprocessing following recent literature guidelines, benchmarking each strategy’s performance. CIRCE implements the algorithm proposed in (Pliner *et al.* 2018) and initially available in the R package Cicero cole-trapnell-lab.github.io/cicero-release. CIRCE runs ∼170 times faster, supports CPU parallelization, and uses significantly less memory. CIRCE allows users to integrate cis-regulatory network computation into scverse workflows (Virshup *et al.* 2023) for all dataset sizes.

We also provide some insights into the impact of input data preparation. Aggregating highly similar cells into ‘metacells’ can mitigate the sparsity and sampling noise inherent to single-cell profiles such as scRNA-seq, while reducing dataset size (Baran *et al.* 2019, Bilous *et al.* 2024). Due to its extreme sparsity, scATAC is often analysed after metacells computation (Pliner *et al.* 2018, Persad *et al.* 2023). We compared cis-regulatory interaction predictions from single-cell versus metacell inputs, as well as binarized versus un-binarized counts. Using promoter capture Hi-C data, we observed higher predictions with CIRCE metacells than Cicero metacells. However, we show that using directly single-cell input data led to even better predictions despite their higher sparsity. While metacells are particularly interesting for condensing information from large datasets and reducing computational requirements, there seems to be a trade-off between resource usage and small performance improvement. CIRCE offers both an improved strategy for metacell computation and the capacity to use single-cell as input up to atlas-sized datasets, by greatly improving processing speed and memory usage.

## 2 Implementation

### 2.1 Workflow and parameters

The methodologies of CIRCE and Cicero differ in both the preprocessing of the scATAC-seq data and the strategy for grouping highly similar cells in metacells before computing the co-accessibility scores. First, Cicero suggests binarizing, then normalizing the count matrix per cell total-count. However, more recent works demonstrate that binarization is not useful for analysing scATAC-seq data (Martens *et al.* 2024), and that scATAC-seq total count normalization can even be deleterious and introduce different biases (Kwok *et al.* 2025). For metacells computation, Cicero proposes by default to compute latent semantic indexing (LSI) reduction from the binarized input data, projecting the cells into a lower-dimensional space of DNA region-topics, summarizing the main chromatin patterns (Pliner *et al.* 2018). Cicero then computes a second dimensionality reduction on this space (UMAP or t-SNE), from which it calculates the nearest neighbours. It has now been demonstrated that t-SNE or UMAP spaces, especially when keeping very few dimensions, do not conserve distance between observations and thus are not adapted to compute nearest neighbours (Chari and Pachter 2023). In CIRCE, we propose by default to group the cells directly on a reduction space generated with LSI, while still providing the option to compute a UMAP reduction and re-use its clustering strategy.

**Figure 1:**
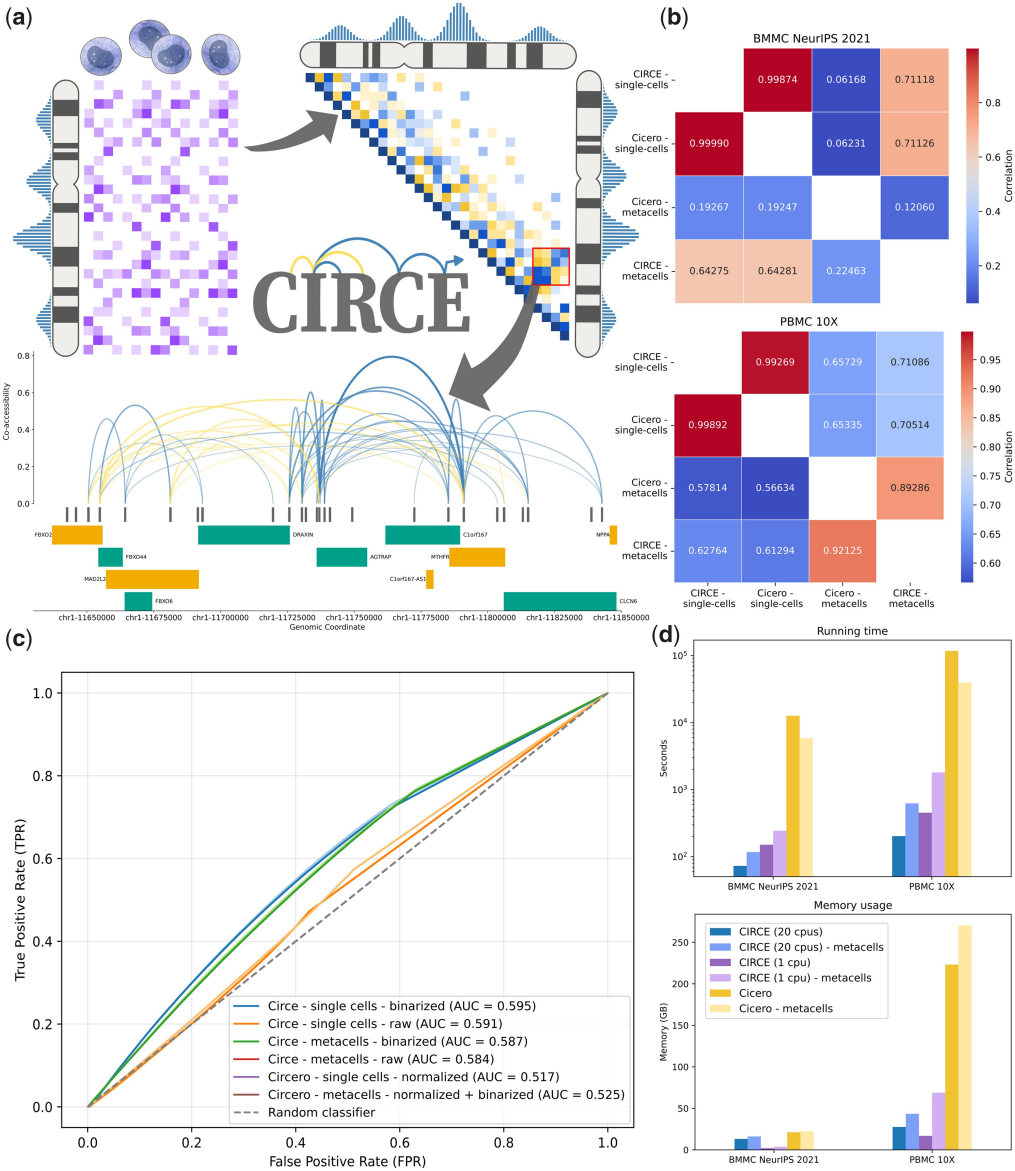
CIRCE workflow and performances. (a) CIRCE workflow overview. From scATAC-seq data, co-accessibility scores are defined as regularized covariance values between DNA regions, using a graphical lasso model and pairwise distance penalties. Cis-coaccessible networks (CCANs) can then be extracted with the Louvain community detection method, as modules of DNA regions with high absolute co-accessibility scores. Co-accessibility scores in a CCAN or a DNA window of interest can then be visualized with different graphical options. (b) Correlation values between the networks obtained from CIRCE, Cicero, and metacells computation on both the BMMC and the PBMC datasets. The upper triangle of the heatmap contains the Spearman correlation, while the lower triangle contains the Pearson correlation. Colour gradient illustrates the correlation values. (c) ROC curves on the recovering DNA region—promoter interactions obtained from the PC-HiC dataset. Different preprocessed inputs are evaluated for each method: CIRCE from raw single-cell counts, binarized single-cell counts, and metacell counts from LSI dimensionality reduction space on both single-cell count matrices, and Cicero on the normalized binarized count matrices and the metacell count matrices obtained from the normalized binarized counts. (d) Running-time and memory-usage of CIRCE and Cicero when running on the single-cell or computing metacells. For CIRCE, we display performances when using multithreading (20 CPUs) and single-threading.

CIRCE relies on the algorithm proposed in (Pliner *et al.* 2018) to compute co-accessibility, which uses Graphical Lasso to impose a distance penalization on the covariance of DNA regions (see Fig. 1a). To reduce computational complexity, these calculations are performed within a sliding window, where the window size corresponds to the maximum distance considered for cis-interactions. This limit is by default 500 kb for human and mouse cis-regulatory interactions in both Cicero and CIRCE, a distance also used as a window limit around promoters to find distal enhancers in several other works (Forrest *et al.* 2014, Stuart *et al.* 2021). Distance penalties are computed between every pair of DNA regions according to the following formula $\rho_{ij}=(1-d_{ij}^{-s})\times \alpha$, with d the distance between two regions.

The scaling exponent *s* of the power-law function estimates the global decay in contact frequency. It has been estimated at 0.75 for the ‘tension globule’ polymer model in human and mouse (Sanborn *et al.* 2015), and a value of 0.85 has been proposed for Drosophila (Sexton *et al.* 2012). The parameter *α*, proportional to the penalty, enforces the sparsity of the final result. As in Cicero, *α* is decided as the lowest value, such as at least 20% of all the pairs across a window are zero entries, ensuring a minimal sparsity in the results, and less than 5% are non-zero entries, ensuring that most connections are short-range. It is estimated by selecting random windows across the genome and averaging alpha values for each of them. To simplify the choice of the species-specific parameters in CIRCE (*s*, window size, distance of long and short-range interactions), users can also specify the organism corresponding to their data to automatically select default literature-based values.

Finally, cis-co-accessibility networks (CCANs) are defined as described in Pliner *et al.* (2018), using the Louvain clustering method (Blondel *et al.* 2008) and the same default parameters.

### 2.2 Implementation details

The most popular Graphical Lasso model (the core of CICERO) in Python is hosted in the scikit-learn (Pedregosa *et al.* 2011) package, but it does not allow a pairwise matrix penalty. Consequently, we use the skggm package (Laska and Narayan 2017), implementing the QUIC algorithm (Hsieh *et al.* 2014) in a scikit-learn compatible package and offering more freedom on model penalization. CCANs identification is based on the Louvain clustering method implemented in the NetworkX package (Hagberg *et al.* 2007).

CIRCE works directly on AnnData objects (Virshup *et al.* 2024), making it fully compatible with any scVerse package (Virshup *et al.* 2023). All results are stored as a sparse matrix for memory usage optimization, directly in the ‘.obsp’ slot of your input AnnData object, and can easily be extracted as a readable table.

Additionally, we provide a tutorial to extend the co-accessible DNA region networks of CIRCE by building prior gene regulatory networks for CellOracle. This tutorial is accessible at https://circe.readthedocs.io/en/latest/examples/4_circe_celloracle_tutorial.html.

## 3 Results and comparison with Cicero

We compared CIRCE and Cicero with and without generating metacells, on a BMMC dataset (1771 cells and 110 235 peaks), and a BPMC dataset (9631 cells and 215 676 peaks). We demonstrated that both implementations were returning near-identical results from the same input, then explored the impact of respective preprocessing strategies on inferring enhancer–promoter interactions.

From the same single-cell ATAC-seq input data, we observed nearly identical results, with respective Spearman and Pearson correlations of 0.9987 and 0.9999 on the BMMC dataset, and 0.9927 and 0.9989 in the PBMC datasets (Fig. 1b). The small difference can be explained by the stochasticity in the algorithm that selects random DNA windows to estimate the parameter *α* (see Section 2).

However, we observed substantial differences when using the respective metacell strategies of CIRCE and Cicero. First, CIRCE’s metacells were still relatively highly correlated with single-cell inferred networks, with respective Spearman and Pearson correlations of 0.71 and 0.64 on the BMMC dataset, and 0.71 and 0.63 on the PBMC datasets. In contrast, the correlations with Cicero’s metacells were lower (Fig. 1b), with particularly high variability between the datasets. On the BMMC dataset, the correlations with the single-cell data run were especially low (i.e. Spearman and Pearson correlations of 0.06 and 0.19, respectively) while the decrease was less marked on the PBMC dataset (i.e. Spearman and Pearson correlations of 0.65 and 0.57).

After observing such differences in the predicted DNA region interactions, we decided to evaluate the networks generated to identify the best strategy. Using a Promoter Capture Hi-C dataset (Javierre *et al.* 2016), we measured how well each method was recovering enhancer–promoter interactions (Fig. 1c). Surprisingly, the networks with the highest AUCs were obtained from the binarized single-cell count matrix and the un-binarized single-cell count matrix (0.595 and 0.591, respectively). In comparison, the predictions from the default corrected counts with Cicero’s function make_atac_cds had an AUC of 0.517. We tested the default metacell strategy of CIRCE on both raw and binarized counts, both leading to a small decrease in the AUC value, with both the binarized and un-binarized metacell counts performing almost identically (0.587 compared to 0.584). The prediction from Cicero’s metacells gave an AUC of 0.525, or a small improvement compared to the predictions from Cicero’s processing of single-cell input. Overall, we observed better enhancer–promoter interaction predictions when using the single-cell matrix directly, and without Cicero’s count correction. This result suggests that Cicero’s count correction is not the best preprocessing for inferring co-accessibility, while simple binarization does not improve the predictions, as already suggested in Martens *et al.* (2024). These modest AUROC values (<0.6) for all networks also reflect the limitations of PC-HiC as a ground truth, as it captures physical contacts rather than coordinated accessibility. While it is widely used for validation (Poirion *et al.* 2024, Zu *et al.* 2023, Murakami *et al.* 2025), the large size of PC-HiC fragments relative to narrow scATAC-seq peaks can inflate false negatives, penalizing the finer-resolution interactions recovered from scATAC-seq.

To further quantify the impact of the different preprocessing strategies on CIRCE predictions, we compared the networks obtained from raw counts, binarized counts, and metacells input (Fig. S1, available as supplementary data at *Bioinformatics* online). In both datasets, we again observed high correlations between networks inferred from raw and binarized counts, whereas both showed lower correlations with networks obtained after Cicero’s count normalization. Additionally, while computing metacells can be interesting for reducing computational time, we observed a small drop in performance with both tested default strategies. In Cicero, a maximum of 5000 metacells is generated; to ensure a fair comparison, we also limited CIRCE to 5000 cells in this evaluation, which might explain the modest decrease in performance.

We also compared running time and memory usage (Fig. 1d) for CIRCE and Cicero on both datasets, with and without computing metacells. On average, CIRCE ran around 170 times faster and used 5.4 times less memory, thanks to both its new implementation and use of multithreading. On the largest dataset (PBMC 10X—single-cell data), CIRCE ran in 3 min 22 s instead of 1 day and 8 h for Cicero, and used 27.6 GB of RAM instead of 223.1 GB. We also report single-threaded runtimes in Fig. 1d, which confirm that CIRCE remains substantially faster than Cicero even without parallelization.

Finally, to demonstrate CIRCE’s scalability and the new possibility it offers, we analysed a human atlas of foetal single-cell chromatin accessibility (Zhang *et al.* 2021). CIRCE processed the 720 616 cells and 1 041 455 DNA regions into a co-accessibility network in 42 min on an HPC. It identified 84 479 373 pairs of co-accessible regions. Since most of the computational time is actually spent in extracting small DNA windows from the initial input (columns of the AnnData input), we recommend using the csc_matrix format of scipy when analysing huge atlases since it is optimized for column extractions.

## 4 Discussion

Here, we present CIRCE, a Python package for predicting cis-regulatory DNA interactions from single-cell chromatin accessibility data. CIRCE offers an optimized implementation of the algorithm proposed in (Pliner *et al.* 2018), updated to address the characteristics of recent large-scale single-cell datasets and compatible with the scverse environment. While providing very similar results to Cicero, CIRCE runs much faster, uses less memory and facilitates the incorporation of cis-regulatory DNA interaction networks in Python workflows.

We also provide new insights into preprocessing strategies for single-cell ATAC and the use of metacells to infer co-accessibility with this algorithm. We reveal that binarization is unnecessary and that count normalization can be counterproductive. While different metacell strategies might improve the results by reducing noise, we did not observe such improvement from the tested strategies. However, they allow preserving good performances with a lower number of observations and reducing their sparsity. CIRCE’s optimized implementation will also permit analysing much bigger datasets without having to reduce the number of cells through aggregation. We anticipated that CIRCE will serve as a useful foundation for future gene–activity inference tools within the Python and scverse environments, particularly approaches that incorporate enhancer–promoter relationships. Indeed, several methods propose considering chromatin accessibility of enhancers and promoters to estimate gene expression (Pliner *et al.* 2018).

## 5 Materials and methods

### 5.1 BMMC NeurIPS dataset

The BMMC multiome used here was generated for the Open Problems challenge (Luecken *et al.* 2021) and is accessible under the GEO accession number GSE194122. We extracted the smallest batch of 1771 cells and kept all the peaks expressed in at least one cell (110 235 peaks).

### 5.2 PBMC dataset

A PBMC multiome dataset was obtained from https://www.10xgenomics.com/datasets/pbmc-from-a-healthy-donor-granulocytes-removed-through-cell-sorting-10-k-1-standard-1-0-0. From the fragment files, we applied the same dataset preparation as in GRETA (Badia-i-Mompel *et al.* 2024). The peak calling and merging were achieved with Snapatac2 (Zhang *et al.* 2024), through the functions snap.tl.macs3, snap.tl.merge_peaks, and snap.pp.make_preaks_matrix. Only the cells expressing more than 100 genes in the scRNA-seq data were then kept. The final dataset contained 9631 cells and 215 676 peaks.

### 5.3 PC-HiC dataset

The promoter capture HiC (PCHiC) dataset used to evaluate DNA region interactions prediction in the PBMC dataset is described in Javierre *et al.* (2016). The processed interaction table is available as ‘PCHiC_peak_matrix_cutoff5.tsv’ in Data S1, available as supplementary data at *Bioinformatics* online: https://ars.els-cdn.com/content/image/1-s2.0-S0092867416313228-mmc4.zip.

### 5.4 PC-HiC processing and overlap evaluation

The PC-HiC interaction dataset, originally provided in hg19 coordinates, was converted to the hg38 genome build by lifting over both DNA regions of each interaction independently. Interactions for which either region failed to map were removed, and the remaining successfully mapped interactions were used as the PC-HiC reference.

Overlap between CIRCE or Cicero predicted links and PC-HiC interactions was assessed by comparing pairs of DNA regions between the two datasets. We considered all pairs between promoters contained in the PC-HiC dataset and regions in a 500 kB window around them. A predicted link was considered to match a PC-HiC interaction when each of its two regions overlapped one of the two regions in the corresponding PC-HiC pair by at least one base pair. Because scATAC-seq peaks are unstranded, the two regions in a predicted link could correspond to the two PC-HiC regions in either order. Overlap between two DNA regions was evaluated using the *bedtools intersect* function, applying its default criterion of requiring at least one shared nucleotide on the same chromosome, with no minimum fractional overlap or window extension.

### 5.5 Foetal human single-cell chromatin accessibility Atlas

The scATAC-seq atlas (Domcke *et al.* 2020), already preprocessed, was downloaded fromhttps://scglue.readthedocs.io/en/latest/data.html under the name Domcke-2020.h5ad.

### 5.6 Reproducibility

The experiments are implemented as a Snakemake pipeline containing all the code to reproduce the experiments at https://github.com/cantinilab/circe_reproducibility. Singularity images for CIRCE and Cicero are available at https://zenodo.org/records/17661273.

All the computations were realized on an HPC equipped with Linux Red Hat 8.8 and 2 AMD EPYC 7552 48-Core processors. For the benchmarking, CIRCE and Cicero were executed from their respective singularity containers (available in the reproducibility repository). Resource usage was limited to 20 cores and 430 GB of RAM through Snakemake rule configuration.

## Supplementary Material

btag092_Supplementary_Data

## Data Availability

The BMMC multiome used here was generated for the Open Problems challenge (Luecken *et al.* 2021) and is accessible under the GEO accession number GSE194122. A PBMC multiome dataset was obtained from https://www.10xgenomics.com/datasets/pbmc-from-a-healthy-donor-granulocytes-removed-through-cell-sorting-10-k-1-standard-1-0-0. The promoter capture HiC (PCHiC) dataset used to evaluate DNA region interactions prediction in the PBMC dataset is described in (Javierre *et al.*, 2016). The processed interaction table is available as “PCHiC_peak_matrix_cutoff5.tsv” in the supplemental data S1: https://ars.els-cdn.com/content/image/1-s2.0-S0092867416313228-mmc4.zip The scATAC-seq atlas (Domcke *et al.*, 2020), already preprocessed, was downloaded from https://scglue.readthedocs.io/en/latest/data.html under the name Domcke-2020.h5ad.
