# Supplementary material for: CIRCE: a scalable Python package to predict cis-regulatory DNA interactions from single-cell chromatin accessibility data
Source: Bioinformatics. 2026 Feb 24;42(3):btag092. doi: 10.1093/bioinformatics/btag092 (PMC12987762; doi:10.1093/bioinformatics/btag092)
Supplement: btag092_Supplementary_Data [file btag092_supplementary_data.zip › Supplementary_materials.docx]

**Supplementary materials**

**Supplementary Figure**

**
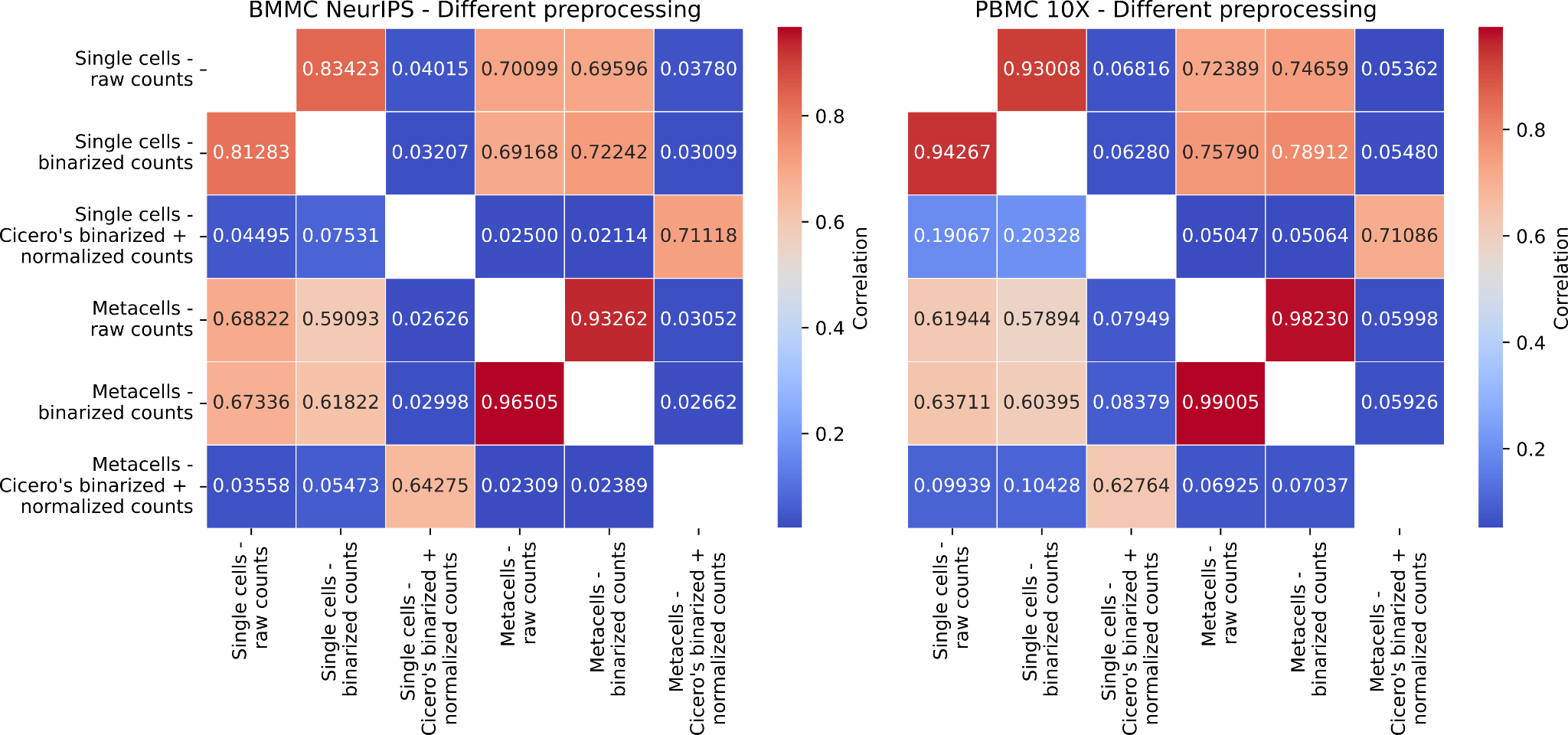
Supplementary Figure 1. Correlation between CIRCE networks from different preprocessing strategies.** Correlation values between the CIRCE networks with three different preprocessing strategies of the count matrices. The upper triangle of the heatmap contains the Spearman correlation, while the lower triangle contains the Pearson correlation. Colour gradient illustrates the correlation values.
