# Supplementary figures and images for: CIRCE: a scalable Python package to predict cis-regulatory DNA interactions from single-cell chromatin accessibility data
Source: Bioinformatics. 2026 Feb 24;42(3):btag092. doi: 10.1093/bioinformatics/btag092 (PMC12987762; doi:10.1093/bioinformatics/btag092)

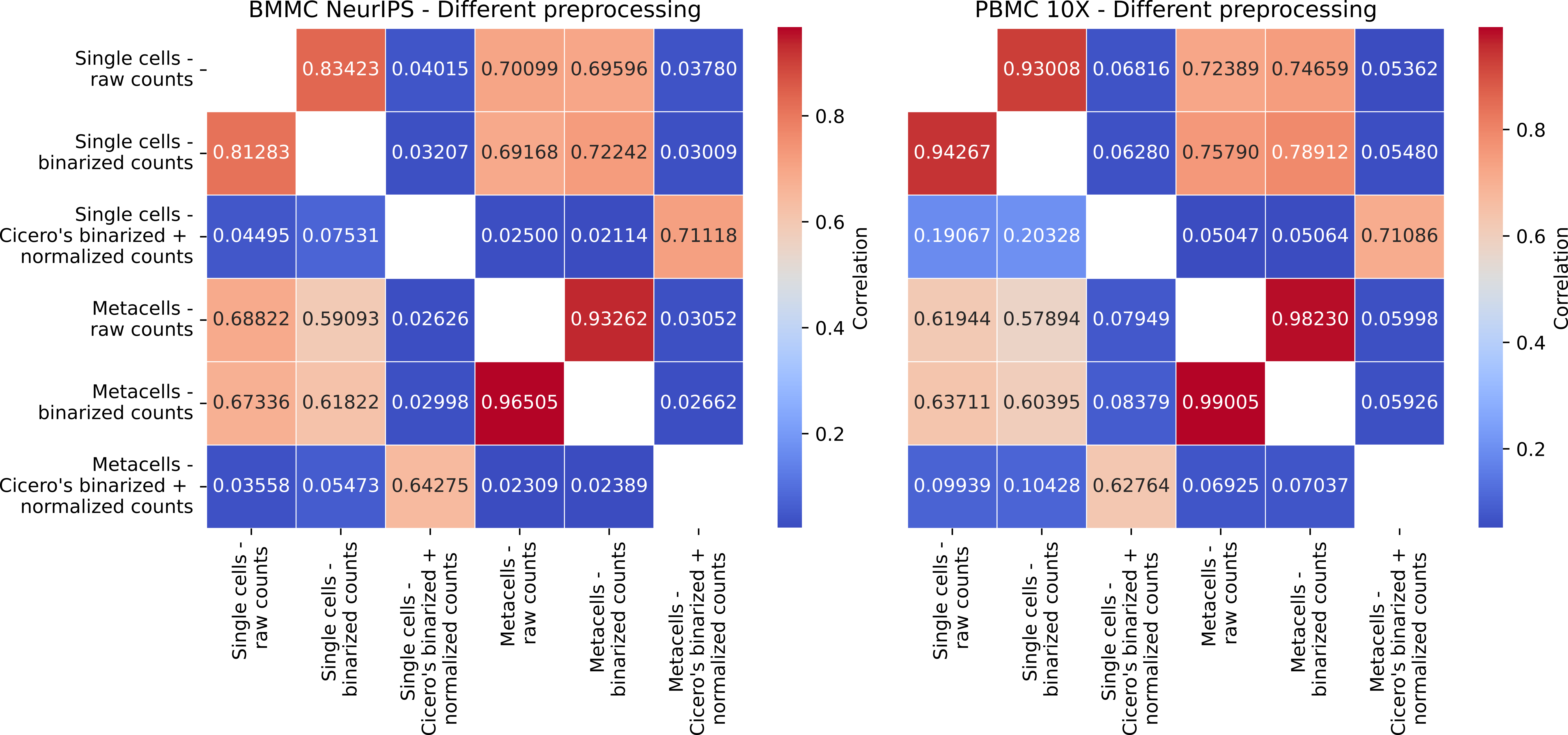

Supplement: btag092_Supplementary_Data [file btag092_supplementary_data.zip › SuppFig1_high.png]
